# Editorial: Pathogenesis of *Leptospira*

**DOI:** 10.3389/fcimb.2018.00322

**Published:** 2018-09-11

**Authors:** Elsio A. Wunder, Azad Eshghi, Nadia Benaroudj

**Affiliations:** ^1^Department of Epidemiology of Microbial Diseases, Yale School of Public Health, New Haven, CT, United States; ^2^UVic Genome BC Protein Centre, Victoria, BC, Canada; ^3^Biology of Spirochetes Unit, Institut Pasteur, Paris, France

**Keywords:** *Leptospira*, leptospirosis, pathogenesis, virulence, regulation, immune response

*Leptospira* are a diverse group of spirochete bacteria classified into 13 pathogenic species and >300 serovars (Picardeau, 2017; Thibeaux et al., 2018). *Leptospira* are the etiological agents of leptospirosis, a neglected life-threatening disease that occurs in a diverse range of epidemiological settings and affecting prominently the world's most impoverished populations (Costa et al., 2015). Leptospirosis causes life-threatening manifestations such as pulmonary hemorrhage syndrome (LPHS) and Weil's disease and has emerged as a major worldwide cause of hemorrhagic fever and acute kidney injury (Ko et al., 1999).

*Leptospira* are highly motile and able to penetrate abraded skin and mucous membranes and cross tissue barriers, which facilitates hematogenous dissemination and systemic infection (Wunder et al., 2016). Infection in susceptible hosts manifests as an acute disease, however, a broad range of mammalian reservoirs can chronically carry the bacteria in their proximal kidney tubules. The bacteria are excreted through the urine and their ability to persist for weeks to months in the environment facilitates the transmission to humans, accidental hosts, coming into contact with *Leptospira* contaminated water or soil (Ko et al., 2009; Picardeau, 2017).

With more than one million cases each year, leptospirosis is a leading zoonotic cause of morbidity and mortality worldwide (Costa et al., 2015). Mortality from severe leptospirosis is high (>10%) despite aggressive supportive care. Diagnosis requires laboratory testing since the clinical presentation of early phase leptospirosis is non-specific and the epidemiological occurrences are foreign to the medical community. Current diagnosis is antiquated which hampers outpatient-based interventions aimed at reducing high mortality and contributes to the under-recognition of the disease (Riediger et al., 2017; Nabity et al., 2018). Limited understanding of leptospirosis determinants has hampered development of new diagnostic and therapeutic approaches. It is therefore essential to focus research on elucidating key virulence factors and pathogenesis mechanisms which will ultimately provide the knowledge needed for better diagnosis and treatment of leptospirosis. This compilation of research and review articles advances our understanding of the features and mechanisms that pathogenic leptospires adopt and explore to successfully establish infection in the host.

Adhikarla et al. described a novel-signaling pathway and the first virulence-associated two-component system (TCS) called *Leptospira* virulence regulator (lvr). This unique *Leptospira* TCS system, controls virulence and motility in pathogenic *Leptospira* and its characterization unveils the existence of a complex signaling network in this genus. Zhukova et al. provided the first genome-wide Transcriptional Start Site (TSS) and promoter maps for the pathogen *L. interrogans*. The authors analyzed the RNA from bacteria cultured under two temperatures, 30° and 37°C, revealing a major conservation of primary TSS at both temperatures. Furthermore, over 500 putative small regulatory RNAs (sRNAs) were identified, with regulatory functions yet to be characterized in this pathogen. These articles provide a framework for understanding how *Leptospira* can reprogram and adapt to the host, and contribute essential information for genetic studies.

da Silva et al. determined that leptospiral extracellular proteases display proteolytic activity against host proteoglycans and plasma proteins, most likely with the participation of metalloproteases. Furthermore, they were able to demonstrate that attenuated and saprophytic species did not display proteolytic activity, indicating that the ability to degrade host molecules correlates with *Leptospira* virulence. Protein secretion in *Leptospira* is a relatively unexplored area of research and this study provided evidence that extracellular proteins of *Leptospira* likely contribute to the pathogenic mechanisms required for infection.

Cagliero et al. scrutinize the cytokine response caused by leptospiral infection and the different ensuing outcomes in susceptible and resistant animals, and how this host response can be used for diagnosis or control of the disease. Similarly, de Castro et al. described the role of the Complement System murine C5, to actively kill leptospires and control their dissemination during the early phases of the disease. Both articles give us important insights about the role of the innate immune system as a first and potentially essential response to *Leptospira* infection.

To answer the fundamental question in regard to the equilibrium between pathogenic leptospires and reservoir hosts, Nally et al. used the dialysis membrane chamber (DMC) (Caimano et al., 2014) in the rat model of chronic infection to identify differentially expressed proteins and post-translational modifications that are associated with the mammalian host signals, including 20 and 7 protein isoforms of LipL32 and LipL41, respectively. Furthermore, Nally et al. developed an inbred immunocompetent rat model to study the pathophysiological pathways involved in the chronic infection. Their results indicate that other than systemic immune response, the local lymphoid organ should be considered when studying renal colonization.

Santos et al. analyzed the whole-genome of 67 strains of *L. interrogans* serogroup Icterohaemorrhagiae, 55 and 12 isolates of serovars Copenhageni and Icterohaemorrhagiae, respectively. Serovars Copenhageni and Icterohaemorrhagiae are recognized to be the most virulent ones among all pathogenic species of *Leptospira*, and responsible for the majority of the reported severe cases. Their results showed that both serovars are closely related but with a distinct spatial clustering. Furthermore, they were able to identify a single indel as the only sequence variation between them with a high discriminatory power to genetically distinguish those two serovars.

Recent breakthroughs in genetic manipulation of *Leptospira* and whole-genome sequencing have provided tools and information to conduct research at the molecular level and better understand the biology and virulence of this unique spirochete. The collection of articles compiled in this research topic is exemplary in advancing our limited understanding of *Leptospira* pathogenesis and this information will support the development of new therapeutic and prevention approaches against this important yet neglected zoonotic disease.

## Author contributions

EW drafted the editorial. NB, AE, and EW contributed to the writing and review process.

### Conflict of interest statement

The authors declare that the research was conducted in the absence of any commercial or financial relationships that could be construed as a potential conflict of interest. The reviewer BA and handling Editor declared their shared affiliation.
